# Polyacrylamide Hydrogel Enriched with Amber for In Vitro Plant Rooting

**DOI:** 10.3390/plants12051196

**Published:** 2023-03-06

**Authors:** Lyudmyla Kernosenko, Kateryna Samchenko, Olena Goncharuk, Natalya Pasmurtseva, Tetiana Poltoratska, Olena Siryk, Oksana Dziuba, Oleg Mironov, Katarzyna Szewczuk-Karpisz

**Affiliations:** 1 F.D. Ovcharenko Institute of Biocolloidal Chemistry, National Academy of Sciences of Ukraine, 01030 Kyiv, Ukraine; 2 Department of Bioenergy, Bioinformatics and Environmental Biotechnology, Faculty of Biotechnology and Biotechnics, National Technical University of Ukraine “Igor Sikorsky Kyiv Polytechnic Institute”, 03056 Kyiv, Ukraine; 3 Institute of Agrophysics, Polish Academy of Sciences, 20-290 Lublin, Poland; 4 M. M. Hryshko National Botanical Garden, National Academy of Sciences of Ukraine, 01030 Kyiv, Ukraine; 5 L.M. Litvinenko Institute of Physical-Organic Chemistry and Coal Chemistry, National Academy of Sciences of Ukraine, 01030 Kyiv, Ukraine

**Keywords:** plant rooting, polyacrylamide gel, amber, acrylamide toxicity, biotesting, *Daphnia magna*

## Abstract

In this work, a new material for in vitro plant rooting based on highly dispersed polyacrylamide hydrogel (PAAG) enriched with amber powder was synthesized and investigated. PAAG was synthesized by homophase radical polymerization with ground amber addition. Fourier transform infrared spectroscopy (FTIR) and rheological studies were used to characterize the materials. They showed that the synthesized hydrogels have physicochemical and rheological parameters similar to those of the standard agar media. The acute toxicity of PAAG-amber was estimated based on the influence of washing water on the viability of plant seeds (pea and chickpea) and *Daphnia magna*. It proved its biosafety after four washes. The impact on plant rooting was studied using the propagation of *Cannabis sativa* on synthesized PAAG-amber and compared with agar. The developed substrate stimulated the rooting of the plants to more than 98% in comparison to standard agar medium (95%). Additionally, the use of PAAG-amber hydrogel markedly enhanced metric indicators of seedlings: root length increased by 28%, stem length—by 26.7%, root weight—by 167%, stem weight—by 67%, root and stem length—by 27%, root and stem weight—by 50%. This means that the developed hydrogel significantly accelerates reproduction and allows obtaining a larger amount of plant material within a shorter period of time than the standard agar substrate.

## 1. Introduction

Usually, the in vitro cultivation and reproduction of plants are carried out in solid nutrient media, and a high-cost agar substrate is the most used in this process [1,2,3,4,5,6,7,8,9]. This material is non-renewable, so for each subsequent passage of plants, it must be used de novo [10]. Because of the agromarket’s needs to propagate plants commercially, the agar substrates are used in large amounts. To avoid such high costs, the search for alternative approaches is demanded. There is a great need to substitute the agar with cheaper disposable materials, such as starch [11] or gum [12], or to develop an environmentally friendly solid substrate with optimal physicochemical and functional properties suitable for multiple uses.

These requirements can be met by hydrogels based on spatially cross-linked hydrophilic polymers such as polyacrylamide, which have the inherent ability to sorb and retain a significant amount of aqueous solutions due to adequate swelling properties. This process leads to an increase in hydrogel volume, but its geometric shape remains unchanged. The so-called “smart hydrogels” are spatially cross-linked hydrophilic polymers with a three-dimensional structure capable of responding to various external physical factors, including minor changes in pH, temperature, ionic strength, etc., by changing the swelling degree, volume, sorption and diffusion characteristics [13,14,15,16]. They can be synthesized in different consistencies and arbitrary shapes, particularly in the form of granules of controlled sizes. Such hydrogels are characterized by high hydrophilicity and biological tolerance, as well as improved optical, sorption, and diffusion properties, which can be adjusted by varying the monomer composition and cross-linking density. The listed peculiarities make them suitable for immobilization of a wide range of both organic and inorganic compounds, as well as for use in novel technologies, namely water treatment [17], manufacturing of selective membranes [18,19], cell cultivation (in particular, stem cells) [19], targeted delivery, and controlled release of anticancer drugs [15]. In recent years, substrates based on such hydrogels have found application for plant vegetation under controlled conditions due to their ability to sorb and slowly release the necessary bioelements into the environment under plant root exudate action [20,21,22,23].

Some natural organic compounds with fungicidal and antibacterial properties can be included in the hydrogels’ structure to enhance their functional properties. Primarily succinic acid and products of its transformation can be used as an additional source of microelements, endogenous physiologically active substances, and growth or cell metabolism stimulators [24,25,26,27]. It is known that amber, in particular, amber chips from the waste of various industries, is an environmentally safe and competitive product, so the inclusion of natural organic compounds in hydrogel substrates can be an effective and cost-effective way to increase the biological activity of the nutrient medium for plants propagation. In addition to the increasing bioactivity issue, the problem of ensuring biosafety is relevant for such materials. Since the monomers that compose the basic structural unit of polymers, and particularly hydrogels, are rather toxic materials and some of them always remain unreacted, the problem of synthesized hydrogel purification from residual microquantities of initial compounds is extremely important. The most affordable and effective way to purify hydrogels is to wash them repeatedly with distilled water. The risk control of toxic compounds in washing water is one of the most important steps in the preparation of synthesized materials for further use.

Today toxicology uses not only traditional spectrometric methods of analysis, but also a variety of biotesting methods that allow obtaining a comprehensive toxicological assessment of the aqueous environment using living test objects, including plants (cereals and legumes, some algae, etc.) and animals (unicellular, crustaceans, worms, etc.). Bioassays involving aquatic organisms, such as *Daphnia magna*, are common and widely used to study the toxicity of various chemicals [28,29]. Biological tests on a biomodel of *D. magna* are standardized in many countries [30]. Short-term testing allows determining the acute toxic effect of compounds in aqueous solution on the survival rate of the branched crustacean *D. magna*, which is one of the most sensitive biomarkers for determining the toxicity caused by different classes of chemical compounds [28,29,30,31].

The aim of this work was to develop an agar substitute for enhancing in vitro plant rooting based on highly dispersed polyacrylamide hydrogels with biologically active amber that meet the requirements of high biocompatibility and suitable moisture content, as well as demonstrating environmental safety.

## 2. Materials and Methods

### 2.1. Materials

Acrylamide (AA) (C_3_H_5_NO, “MERCK”, Darmstadt, Germany); N,N′-methylenebisacrylamide (MBA) (C_7_H_10_N_2_O_2_, “MERCK”, Germany); potassium persulfate (K_2_S_2_O_8_, “SIGMA”, USA); and sodium metabisulfite (Na_2_S_2_O_5_,”SIGMA”, St. Louis, MO, USA) were applied in the study without additional purification. Double-distilled water was used as a solvent in all experiments.

For the synthesis of modified polyacrylamide gel (PAAG), samples of Ukrainian amber from the Zhytomyr, Olevsk and Rivne regions (Klesovo and Volodymyrets-Vostochny fields) were used.

### 2.2. Methods

#### 2.2.1. Synthesis of PAAG

The method of homophase radical polymerization in aqueous medium was used for synthesis of hydrogels based on acrylamide [32]. Polymerization was carried out at room temperature. The monomer solution was stirred on a laboratory magnetic stirrer (MM-5, 1200 rpm), then the redox initiation system (potassium persulfate–sodium metabisulfite) was added. The components’ concentrations in the initiating mixture were chosen to achieve complete polymerization within 1 h and to prevent excessive composition heating, which could adversely affect the hydrogels’ properties. Cross-linking with the spatial network formation occurred due to copolymerization with a bifunctional monomer, N,N′-methylene-bis-acrylamide (MBA). The gel was dispersed in a mortar to a granule size of Ø1–2 mm. The components used for PAAG synthesis are summarized in Table 1.

The synthesized PAAGs were repeatedly washed to remove unreacted components of the reaction mixture in distilled water in a ratio of 1 to 50 at a temperature of 45 °C for 7 days. The washing process was monitored spectrophotometrically using a UV spectrophotometer SPECORD M40 (Carl Zeiss) [32]. The washing water was further analyzed for the presence of toxic impurities using test objects of plant and animal origin.

#### 2.2.2. Modification of PAAG with Amber

The amber was ground using the laboratory mill Kinematica AG (Polymix^®^ PX-MFC 90 D), and then it was divided into fraction sizes of from 2–3 to15–20 mm. Milled samples of amber were pre-washed in running water and 10% NaCl. After that, the amber samples were cleansed of NaCl residues and dried at room temperature for several days or in a drying cabinet at 40–50 °C. Amber samples were pre-cooled with liquid nitrogen to prevent the destruction of natural components during mechanical grinding due to overheating. Two types of amber were used: raw amber (sample A1) and amber crumb (sample A2). The amber crumb is a cheap raw material (remains as jewelry production waste), the use of which allows obtaining a substrate with a biologically active component but also effectively solves the issue of disposal of valuable raw materials.

Synthesis of polyacrylamide gels containing amber and cleansing them of unreacted components were performed according to the method described above. The fine amber A1 and A2 were added to the reaction mixture at a ratio of monomers and amber of 20 to 1 (Table 1). The obtained materials were also washed with water to control the release of amber incorporated in the hydrogel structure. During this process, no leaking of amber particles or a change of hydrogel color was observed. The UV spectra obtained for the washing waters did not contained additional peaks. This is why it was stated that amber is firmly fixed in the hydrogel structure and is not washed away by water.

#### 2.2.3. Biotesting of Acrylamide Toxicity Using Pea and Chickpea Seeds

Biotesting of the toxicity of washing waters was performed by the Nelyubov method [33], which is based on the fact that dyes stain only dead cell plasma (the plasma of a viable cell remains unstained). The viability was determined using pea and chickpea seeds. According to this technique, the seeds were soaked and, after 8 h, were released from the seed coat with a needle without damaging them. Ten peas were placed in aqueous solutions of acrylamide with a concentration ranging from 0.001% to 10% and kept at a temperature of 30 °C for 3 h. Then, the peas were transferred to 0.2% indigo carmine aqueous solution and kept for the next 3 h, after which the dye was drained, the peas were washed with distilled water, and their viability was determined according criteria described in [33]. Biotesting of washing waters (from the 1st to the 7th washings) was carried out similarly.

#### 2.2.4. Bioassay Using *Daphnia magna*

The acute toxicity of polyacrylamide hydrogel was also determined using the model of the hydrobiont *D. magna* (according to ISO 10706:2000 [34]). This method is based on estimating the influence of aqueous solutions on the *D. magna* mortality rate (%). *D. magna* was kept in ventilated aquariums with carbon-filtered tap water (pH = 7.3 ± 0.3) at a temperature of 18–22 °C and a dissolved oxygen concentration >6.0 mg/L [34]. The illumination of the cultivation was 400–600 lux with a light period of 16 ± 1 h, and 8 ± 1 h of darkness. The water from the 1st to the 7th washings with an acrylamide concentration of 0.00125 to 0.00001 mmol/L were used for cultivation. Distilled water was used as a control. The experiment used newborns aged 12–24 h, obtained by cultivation. In each 50 mL glass container with 30 mL of the test solution, seven individuals of *D. magna* were placed. The newborn *daphnias* were fed using *Chlorella vulgaris* or a suspension of baker’s yeast 2 h before the experiment and were not fed during the experiments. The mortality of individuals in each beaker was assessed within 24 to 48 h. Specimens that moved freely in the water column or floated to the tank surface no later than 15 sec after light shaking were considered alive. The experiments were performed in triplicate. The sensitivity of *D. magna* to the reference model toxicant, potassium dichromate (K_2_Cr_2_O_7_), was also determined (for 24 h). This allowed us to assess the suitability of *D. magna* culture for biotesting.

#### 2.2.5. Investigation of Hydrogel Structure and Rheological Properties

Fourier transform infrared spectroscopy (FTIR) spectra of powdered samples over the 4000–400 cm^−1^ range were recorded using a ThermoNicolet iS10 FTIR spectrometer with a diffuse reflectance mode.

The rheological properties of PAAG were investigated by a rotary viscometer Rheotest 2.1 using a cylindrical system Z in the range of shear rates from 2.43 to 1073 s^−1^ at a temperature of 20 °C. For the investigation of rheological properties, the synthesized, chemically cross-linked PAAG samples were pre-treated in a ball mill with subsequent sieving to obtain a fraction of gel particles d <1 mm in the non-swollen state. Then, the dry gel particles were mixed with a given amount of water, which corresponded to the conditions of their use for the in vitro rooting of plants.

#### 2.2.6. In Vitro Plant Rooting

Glesia industrial hemp (*Cannabis sativa*) was selected as a test object. The used variety of monoecious non-narcotic hemp with dense rhomboid inflorescences and seed productivity provides the ability to produce a seed yield of 2.0–2.2 t/ha. The period from emergence to the onset of the phase of technical maturity is 88-93 days, and to the onset of the phase of biological maturity, 100–120 days. The plant material was obtained from the Institute of Bast Crops of the National Academy of Agrarian Science of Ukraine. Cuttings of juvenile plants were the primary materials for obtaining sterile (microorganism-free) hemp plants in vitro. Grafting of plants was performed in the presence of three internodes on plants in culture vessels. The cuttings were pre-treated with 70% ethanol for 1 min, followed by treatment with 0.5% thimerosal (C_9_H_9_HgNaO_2_S) for 1.5 min.

Parts of the stem (micropub) with two axillary buds on the basal part were placed vertically in agar and hydrogel substrates to a depth of 0.5–1 cm. Each vessel contained one 3 cm two-node explant of uniform size. Explants were cultured in sterile glass vessels (8 × 14 cm) and capped with nonventilated lids. Culture vessels were autoclaved at 121 °C and 100 kPa for 20 min.

Cultivation was performed on solid substrates: agar-agar (control) and hydrogel substrates (PAAG and PAAG-A2), each saturated with Murashige-Skuga (MS) culture medium with the concentration of macronutrients reduced by half. This medium contained (1/2 MS) 0.5 doses of macro- and micronutrients with the addition of 30 g/L sucrose and had a slightly acidic reaction (pH 5.6–6.0) [35]. No antibiotics or plant growth regulators were used. Hydrogels were mixed with MS medium in a 1:10 weight ratio. For comparison, the agar-based (7.45 g/L) medium with the same MS and sucrose content was used. The complete cultivation cycle was 60 days. The first stage of introduction to the culture took place at the air temperature of 26–28 °C. The obtained specimens were subcultured on MS medium under illumination with fluorescent lamps (2000–2500 lux) with a 16 h photoperiod at a temperature of 24–26 °C and a humidity of 70%.

In order to evaluate the allelopathic activity of aerial parts of hemp *Cannabis sativa* L. extracts [36], bioassays were employed. In this method, one-day-old seedlings of cucumber *Cucumis sativus* L. cv. Konkurent were used as a test object [37].

Data were statistically analyzed using STATISTICA 13.0 software (StatSoft, Tulsa, OK, USA). The experiment was conducted randomly with 10 replications. Two experiments were conducted separately for each type of explant. The mean of replications was used for statistical analysis. Mean separation was performed using a multiple range test at 0.05 probability level.

## 3. Results

### 3.1. FTIR

**Figure 1:**
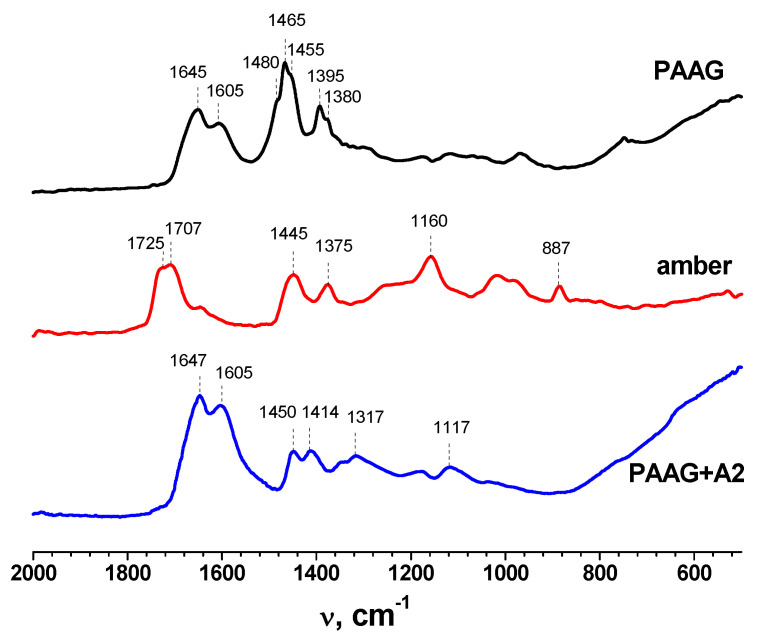
FTIR spectra of amber, polyacrylamide hydrogel (PAAG) and polyacrylamide hydrogel with amber (PAAG−A2).

The FTIR spectra of dried initial PAAG gel, initial amber, and PAAG-gels with amber are given in Figure 1. The most informative peaks of all studied materials were in the range of 1800–800 cm^−1^. The spectrum of PAAG showed two bands at 3436 and 2924 cm^−1^, which corresponded with the N–H stretching vibration of the NH_2_ group and C–H stretching vibrations. The bands at 1645 and 1465 cm^−1^ were attributed to the stretching of the C=O group in amide and CH_2_ scissoring, respectively [38]. In the FTIR spectrum of initial amber, the wide shoulder of the 1160 cm^−1^ peak stretching to 1260 cm^−1^ (known as “Baltic shoulder”) corresponded to the high content of succinic acid and other succinate compounds. The intense peak at ~1700 cm^−1^ was characteristic of the C=O group of carboxylic acids. The bands at 1445 and 1375 cm^−1^ were attributed to C–H symmetric and asymmetric stretching vibrations. The 887 cm^−1^ band could be assigned to the out-of-plane aromatic C–H bending [39,40].

In the spectra of polyacrylamide hydrogels with amber, the bands of amide I (1647 cm^−1^) and amide II (1605 cm^−1^) of acrylamide had the highest intensity. The bands in the range of 3000–3500 cm^−1^ corresponded with symmetric and asymmetric vibrations of amino groups ν_(NH)_ of polyacrylamide. The maximum at 2938 cm^−1^ was attributed to stretching vibrations of the methylene group. The intense maximum ν_(C=O)_ at 1647 cm^−1^ (Amide I) corresponded with the amide fragment, which overlapped with the bending vibration maximum δ_(NH2)_ at 1605 cm^−1^ (Amide II) with the formation of a broadened doublet. In the “fingerprint” region, a doublet at 1450–1410 cm^−1^ caused by bending vibrations of the CH group, as well as a broadened band at ν_(CN)_ = 1350 cm^−1^ (Amide III), were noted [38,41].

### 3.2. Acute Toxicity

**Figure 2:**
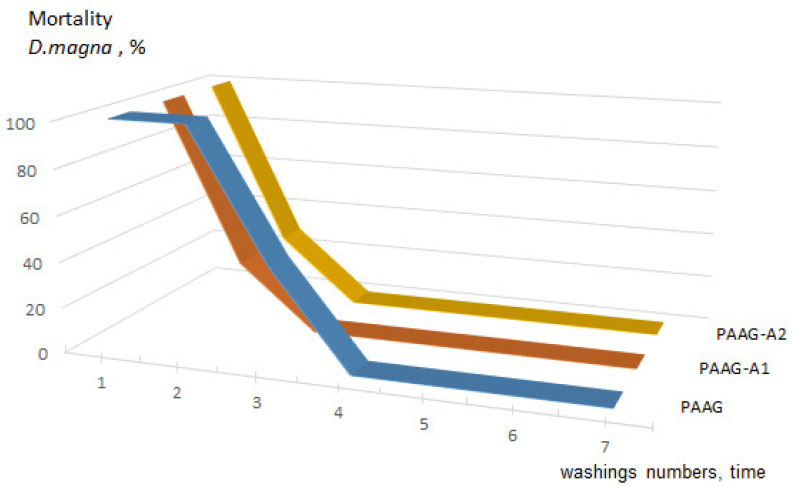
Dynamics of mortality of *D. magna* individuals depending on the number of washings: PAAG—homopolyacrylamide gel; PAAG-A1—homopolyacrylamide gel containing 5.8% raw amber; PAAG-A2—homopolyacrylamide gel containing 5.7% amber crumb.

Synthetic hydrogels, created by polymerization of highly toxic monomers such as acrylamide, contain unreacted residues that should be removed before using them for medical and biological purposes [32]. Acute toxicity of the studied compounds of different washes (from the 1st to the 7th) was determined based on the mortality rate (%) of *D. magna*. Data on the survival of individuals in each sample within 24 and 48 h of exposure are presented in Figure 2 and Table 2.

The performed experiments indicated that after the first wash of all tested samples, complete death of organisms (100%) was already recorded during 24 and 48 h of the experiment. However, the mortality decreased to 28.6% and 57.2% during 48 h in the second washing water for PAAG-A2 and PAAG-A1, respectively (Figure 2). In the third washing water, the mortality of *D. magna* individuals in the PAAG-A1 and PAAG-A2 samples was absent. No cases of mortality were recorded during 24 and 48 h of the experiment. Based on these results, *D. magna* was considered as an organism of high sensitivity to washing water composition, which could be used for diagnosis and risk assessment of the hydrogels and unreacted compounds.

**Figure 3:**
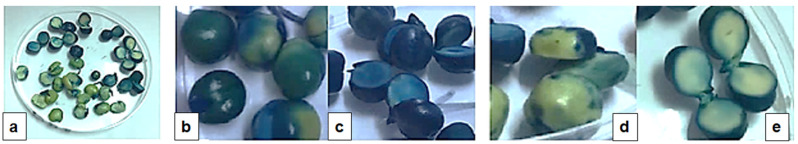
Visualization of Nelyubov method used for determining the viability of peas (**b**,**d**) and chickpeas (**c**,**e**): (**a**) the control (distilled water); (**b**,**c**) the first washing; (**d**,**e**) the second washing. The incubation time was 90 min.

The results of biotesting of the toxicity of washing water by the Nelyubov method are depicted in Figure 3. It was found that 1–3 washes of hydrogels, in which the concentration of acrylamide ranged from 0.00054 mol/dm^3^ to 0.00125 mol/dm^3^ (according to the results of measurements using a UV spectrometer), were the most toxic for all tested legumes. For peas, 10% of seeds died, while for chickpeas, 20%. The results of both biotesting experiments are presented in Table 2. They indicated that the washing water from the 4th wash was safe for both *D. magna* and legumes. The hydrogels, washed in this way, can be safely used in practice. Tucson et al. also washed PAAG for 3 days (changing the water every 24 h), after which the gel was safe for further use as a substrate for the study of bacteria [42].

### 3.3. Rheological Properties

The structural and mechanical characteristics of hydrogel composites largely determine the application possibilities of these systems. They are related to bioavailability and release of biologically active components from hydrogel materials. Gels are structured systems that demonstrate the structural and mechanical properties of both liquids and solids. Hydrogel, as a dispersed system, acquires the properties of a solid body, that is, shear modulus and elasticity. The most important rheological characteristics of hydrogels include shear stress and viscosity. Effective viscosity is a characteristic of the equilibrium state between the processes of destruction and recovery. Its fluctuation causes a change in the coagulation-crystallization structure of the hydrogel, affecting its performance characteristics. The spatial structure in the hydrogel is determined by measuring the mechanical properties and, in particular, shear deformation under constant stress. Solids are characterized by a sharp change in the pattern of shear deformation ε depending on the magnitude of the shear stress ***Р***. At rather low stresses (less than the yield strength ***P_k_***), a free flow with constant and extremely high viscosity ***η_1_*** is observed. In this case, the coagulation structure is destroyed, but has time to recover. As the shear stress increases to the yield strength ***P_k_***, the viscosity decreases significantly, down to the lowest limit value ***η_m_***.

**Figure 4:**
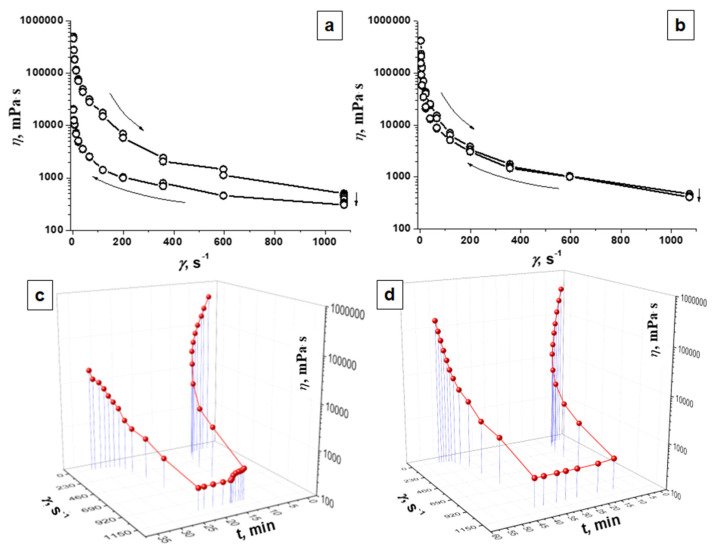
Dependence of effective viscosity (*η*, mPa · s) of polyacrylamide gel (PAAG) (**b**,**d**) and agar gel (**a**,**c**) (**a**,**b**)—on the shear rate (*γ*, s^−1^); (**c**,**d**)—on the shear rate (*γ*, s^−1^) over time (t, min).

Rheological behaviors observed for agar gel and PAAG are presented in Figure 4. Non-linear change in effective viscosity values indicated non-Newtonian system properties of the studied gels. During the measurement, the destruction of interparticle bonds progressed as the shear rate (***γ***) increased, which was manifested by peculiarities in the shapes of the flow curves, causing deviations from straight lines. Measuring in the reverse mode indicated recovery of the effective viscosity due to the restoration of the system structure, but the effective viscosity values remained lower than the initial ones. Both dispersed PAAG and agar gel showed thixotropic properties, as evidenced by the hysteresis loops of the dependence of the effective viscosity of gels on the shear rate (Figure 4a,b).

Table 3 and Figure 4 show the initial and final values of the effective viscosity at the minimum and maximum shear rates for homopolyacrylamide and agar gels. The initial effective viscosity values were close for both gels. The viscosity values at a shear rate of 2.45 s^−1^ at the end of the measurement in the reverse shear rate reduction mode for PAAG and agar were 156.398 and 200.32 Pa∙s, which were 37.0 and 40.2% of the initial value, respectively. This means that PAAG had rheological properties and effective viscosity values similar to those of agar gel.

### 3.4. Plant Rooting

For rooting of plants in vitro, all synthesized hydrogels were saturated with a ½ MS culture medium with a complex of micro- and macronutrients. This process provided the formation of hydrogel composites with sparingly soluble bioelements, which were localized in the hydrogel pores and could gradually diffuse into the external environment. The experiment showed the acceleration of the rooting time of cuttings on PAAG-A2 gel, which was less than 2 weeks of incubation. On agar-agar, this time equaled 3 weeks. In addition, an intensification of growth and development of the main shoot was observed on the hydrogels: 10 and almost 30% higher on PAAG and PAAG-A2, respectively, compared to the samples grown on agar-agar.

**Figure 5:**
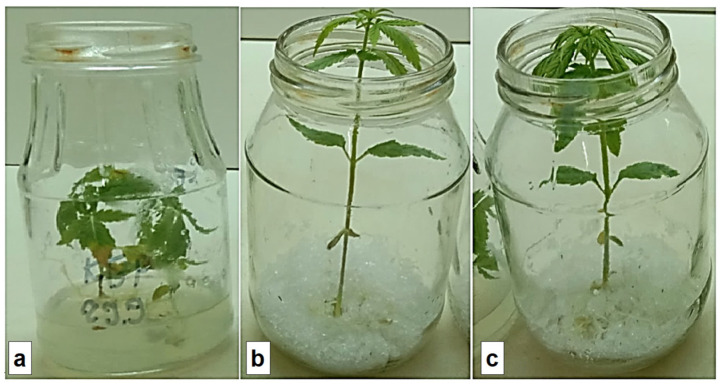
Visualization of the first cultivation cycle of *Cánnabis satíva* (14 days) on: (**a**) agar substrate; (**b**) polyacrylamide (PAAG); and (**c**) polyacrylamide substrate with the addition of amber (PAAG-A2). Each vessel contained one plant.

The rooting of the plants on agar was 95%, while on hydrogel substrates, it was higher than 98%. On hydrogel substrates with the addition of amber, there was a 1.7-fold increase in shoot growth intensity, and on substrates without amber—1.5 fold. When the plants remained for 10–15 days in a tall vessel, their height reached 110–130 mm (Figure 5).

In vitro deposition was carried out with periodic transplantations to the substrate to avoid drying out and changes in the composition of the environment due to the effect of the products of plant metabolism. After the first cycle, the spent hydrogel material was regenerated—washed in distilled water, sterilized, and used in repeated cultivation cycles.

**Figure 6:**
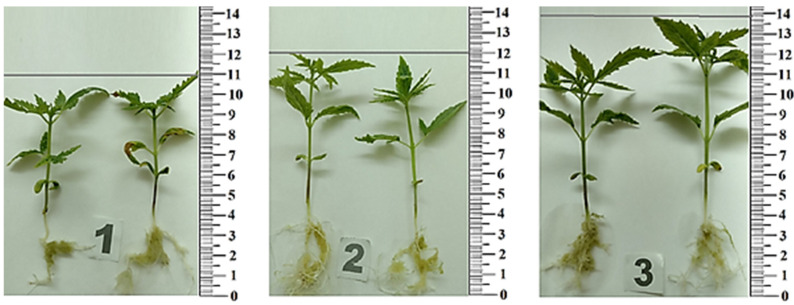
Metric indicators of *Cánnabis satíva* seedling growth: (**1**) on agar substrate; (**2**) on polyacrylamide substrate (PAAG); (**3**) on polyacrylamide substrate with amber addition (PAAG-A2).

Increases in the number of metric indicators of leaf blades were also found. It was found that the dose-dependent effect of plant extracts on test subjects persisted (Figure 6, Table 4). In general, using a hydrogel instead of agar stimulated the growth of *Cánnabis satíva*. Use of hydrogel-amber substrate increased metric indicators of seedlings (in comparison to agar): the root length increased by 28%, stem length—by 26.7%, root weight—by 167%, stem weight—by 67%, root and stem length—by 27%, and root and stem weight—by 50%.

**Figure 7:**
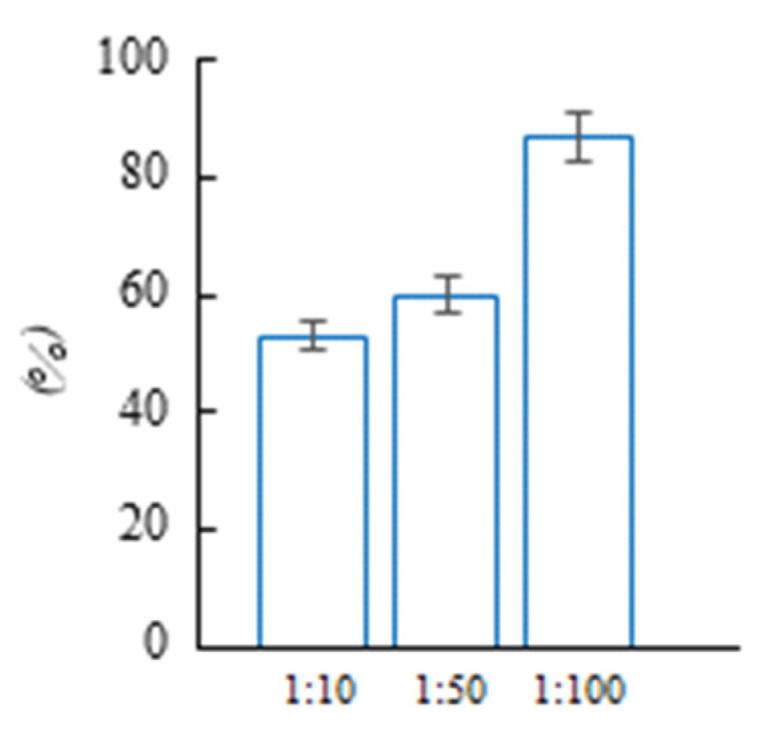
Allelopathic effect of aqueous extract of aboveground parts of *Cánnabis satíva* on seedlings of *Cucumis sativus L*.

In addition, the allelopathic activity of extract from biological material in 1:10, 1:50, and 1:100 dilutions was studied. It was shown that allelopathic stress is dose-dependent when a typical MS medium is used. Under 1:10 dilution, *Cucumis sativus* L. growth inhibition of up to 53% was observed; however, growth inhibition reached 87% (Figure 7). Under 1:100 dilution, the “suppressive” effect showed almost no decrease (95%).

Using acrylamide hydrogel with amber enabled obtaining plant material in a shorter period of time, accelerated the transition of plants from the juvenile to the reproductive phase of development, and increased the intensity of growth and development of the main shoot. The synthesized hydrogel could be used repeatedly after washing, in contrast to agar.

## 4. Conclusions

A spatially cross-linked polyacrylamide hydrogel with immobilized amber was synthesized. It was demonstrated that in terms of its physicochemical and rheological properties, the obtained material is similar to agar-agar and can be used as its substitute for in vitro rooting of plants. It was biosafe after four washings. During each wash, all unreacted toxic monomers and initiator residues were removed from the hydrogel structure; that is, it was purified effectively. The biosafety of the new hydrogel was confirmed by experiments performed using both biological objects, such as pea/chickpea seeds and *D. magna*, and traditional UV spectroscopic methods. There was no mortality of both legume seeds and *D. magna* after the application of water from the 4th washing. The use of the new hydrogel, instead of agar, stimulated the growth of *Cánnabis satíva*. For example, the root and stem weights increased by 167% and 67%, respectively. 

The advantages of the developed composite substrate over agar are: (i) a significant improvement in efficiency for rooting, (ii) the possibility of repeated use, (iii) the amber particles immobilized in the structure of the hydrogel allow achieving the prolonged release of bioactive compounds that stimulate plant growth, which is not possible when using an agar substrate. Therefore, taking into account the above, the described material should be considered as a novel effective substrate for in vitro plant rooting, which can accelerate reproduction and allow obtaining a higher amount of plant material within a shorter period of time in comparison with the agar.

## Figures and Tables

**Table 1:** Composition of reagents used for synthesis of PAAG samples.

Sample	Water, g	AA, g	MBA, g	Amber, %
PAAG	13.6	32	0.04	-
PAAG-A1	11.1	32	0.04	5.8
PAAG-A2	11.6	32	0.04	5.7

**Table 2:** Overall risk assessment of toxic compounds present in washing waters on the viability of biomarkers of plant (legume) and animal (*D. magna*) origin.

Washing/AA Solution	Conc. AA, mmol/L	Death Toll, %					
		PAAG		PAAG-A1		PAAG-A2	
		Daphnia	Peas/Chickpeas	Daphnia	Peas/Chickpeas	Daphnia	Peas/Chickpeas
AA solution	1.4	100 *	80/80	100 *	80/80	100 *	80/90
AA solution	0.14	100 *	40/70	100 *	40/80	100 *	50/70
AA solution	0.014	100 *	40/70	100 *	40/80	100 *	50/70
1	0.00125	100 *	10/20	100 *	10/20	100 *	10/20
2	0.00096	100 **	10	28.6 **	10	28.6 **	10
3	0.00054	42.8 **	10	0	10	0	10
4	0.00007	0	0	0	0	0	0
5	0.00003	0	0	0	0	0	0
6	0.00002	0	0	0	0	0	0
7	0.00001	0	0	0	0	0	0
Control (DV)		0	0	0	0	0	0

* Died on the first day of the experiment. ** Died on the second day of the experiment.

**Table 3:** Effective viscosity at minimum (*γ* = 2.45 s^−1^) and maximum (*γ* = 1073 s^−1^) shear rates.

Sample	*η* (at *γ* = 2.45 s^−1^), Pа∙s		*η* (at *γ* = 1073 s^−1^), Pа∙s	
	Initial	Final	Initial	Final
Agar	498.519	200.32	0.501	0.306
PAAG	422.986	156.398	0.471	0.410

**Table 4:** Metric indicators of *Cánnabis satíva* seedlings.

Experiment Techniques.	*Cánnabis satíva* Seedlings	Root Length (cm)	Stem Length (cm)	Root Weight (g)	Stem Weight (g)	Root and Stem Lengths (cm)	Root and Stem Weight (g)
In vitro	Agar	3.5 ± 0.2	7.5 ± 0.4	0.3 ± 0.05	0.6 ± 0.02	11 ± 0.5	0.9 ± 0.07
	PAAG	4 ± 0.3	8 ± 0.6	0.6 ± 0.03	0.7 ± 0.02	12 ± 0.6	1.3 ± 0.05
	PAAG-A2	4.5 ± 0.3	9.5 ± 0.6	0.8 ± 0.07	1 ± 0.05	14 ± 0.7	1.8 ± 0.06

## Data Availability

All data will be available upon reasonable request from the corresponding author.
